# Valorization Pathway for Grape Pruning and Pomace Waste from the Wine Industry: Energy and Non-Energy Applications

**DOI:** 10.3390/molecules30112332

**Published:** 2025-05-27

**Authors:** José R. Ayala, Benjamín A. Rojano, Marcos A. Coronado, Andrés Felipe Alzate-Arbeláez, Carlos A. Sagaste, Angie D. Vélez, Daniela G. Montes

**Affiliations:** 1 Instituto de Ingeniería, Universidad Autónoma de Baja California, Calle de la Normal S/N Insurgentes Este, Mexicali 21280, BC, Mexico; ramon.ayala91@uabc.edu.mx; 2 Laboratorio de Ciencia de los Alimentos, Facultad de Ciencias, Universidad Nacional de Colombia, Sede Medellín, Calle 59 No. 63-20, Medellín 050034, Colombia; brojano@unal.edu.co (B.A.R.); afalzatea@unal.edu.co (A.F.A.-A.); advelezv@unal.edu.co (A.D.V.); 3 Facultad de Pedagogía e Innovación Educativa, Universidad Autónoma de Baja California, Av. Monclova Esquina con Rio Mocorito s/n, Mexicali 21360, BC, Mexico; carlos.sagaste@uabc.edu.mx

**Keywords:** antioxidant capacity, grape pomace, grape pruning

## Abstract

Wine is a popular beverage worldwide, and its consumption continues to rise, leading to waste, particularly from vine prunings and grape pomace. The aim of this study was to create a valorization pathway utilizing these waste materials. To achieve this, proximate analysis, chemical composition, ultimate analysis, thermogravimetric analysis (TGA), and other physicochemical parameters for both vine prunings and grape pomace were assessed. Based on the results, vine prunings were identified as suitable for direct combustion in energy applications, and grape pomace was found to be suitable as an antioxidant in vegetable oil. Grape pomace extract showed the following results through UV-vis spectroscopy: total phenolic content of 1688.10 mg GAE/100 g, total flavonoids of 1330.39 mg catechin/100 g, and total anthocyanins of 12.61 mg cyanidin-3-glucoside/100 mg. The antioxidant capacity was measured through various assays: FRAP yielded 2179.19 mg ascorbic acid/100 g; DPPH measured 1704.41 µmol Trolox/100 g; and ABTS showed 48,271.31 µmol Trolox/100 g. The ORAC results, as determined by fluorescence spectroscopy, were 53,694.93 µmol Trolox/100 g. HPLC profiling revealed cyanidin as the main anthocyanin (26.52 mg/L) and epicatechin as the most abundant flavonoid (214.29 mg/L). Finally, the antioxidant capacity of grape pomace in sunflower oil was evaluated using OSI. It increased the oil’s stability by up to 42.5%, positioning grape pomace extracts as a source of natural antioxidants in vegetable oils.

## 1. Introduction

The agroindustry is a crucial segment of the industrial sector focused on the production, transformation, storage, and commercialization of agricultural products. Key products processed in this sector include fruits, vegetables, roots, seeds, leaves, tubers, and pods. These products are either sold fresh or processed into various forms, such as nectars, juices, jams, salads, flours, oils, wines, powdered concentrates, and preserves. The grapevine *Vitis vinifera* is a significant woody plant, continuously undergoing cultivation advancements. Grapes, the fruit of the grapevine, grow in clusters and are highly valued [1]. This species belongs to the *V.* genus within the Vitaceae family, encompassing approximately 600 species of climbing shrubs that bear berry-like fruits. Optimal growing conditions for grapevines include regions with adequate rainfall, warm summers, and mild winters [2].

**Figure 1:**
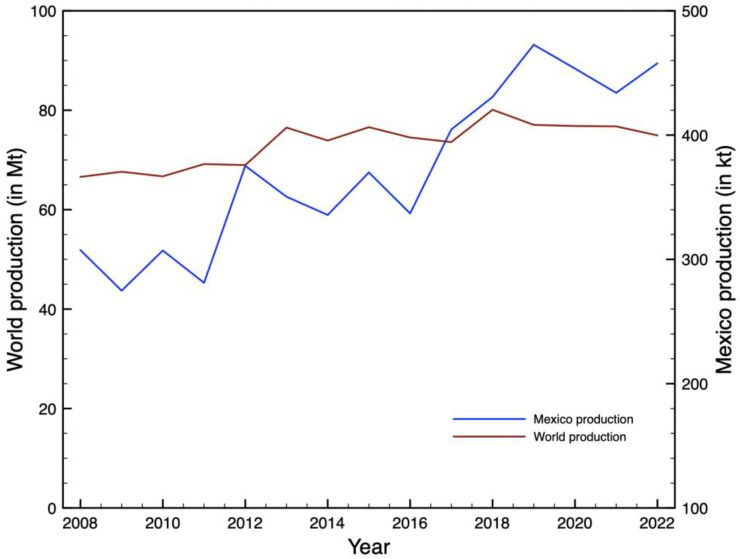
World and Mexico grape production from 2010 to 2022, with data from FAO and SIAP [4,5].

In the wine industry, it is estimated that over 20% of wine grapes become pruning residues [3]. Grape cultivation is among the most significant agricultural activities worldwide, with a global production of 74.94 million tons in 2022 [4]. The leading producers were China with 16.86%, Italy with 11.25%, France with 8.27%, Spain with 7.87%, and the USA with 7.16% [4]. In the same year, Mexico contributed 457,752 tons, accounting for 0.61% of the total world production [5]. Figure 1 illustrates the historical trend from 2010 to 2022 for both global grape production and Mexico’s grape production.

Most of the total grape production (75%) is dedicated to winemaking. Vine plants have the following composition: the trunk and arms make up 38%, the roots 33%, the fruit 15%, the branches 9%, and the leaves 5% [6]. During grape production and processing, byproducts known as vine prunings are generated. Vine prunings are slender, flexible, and knobby stems from which leaves and grape clusters emerge. These shoots result from cutting the vine plants after harvest to control their natural growth, improve yield, and enhance grape quality [3,7]. After the grapes are processed, a paste composed of stems, grape skins, and seeds, referred to as grape pomace, remains [8]. This pomace accounts for approximately 20% of the grape’s weight and is the most significant byproduct of winemaking [9,10]. Because of the nature of the production process, grape pomace is recognized as a significant source of bioactive compounds, including unsaturated fatty acids, vitamins, antioxidants, phenolic acids, flavonoids, tannins, carotenoids, and anthocyanins, among others [11,12].

Valle de Guadalupe is the most important wine region of Mexico, accounting for more than 90% of the national wine production and 75% of national wine sales [5]. In 2023, this region alone had 4708 hectares of cultivated grapevines, with a total production of 27,155.57 tons [5]. There are 150 wineries registered in the region, from local brands to international brands, with a total production of 12.7 million liters in 2022 [5]. Each September, the vineyards are harvested, which produce a great variety of waste materials, including vine pruning and grape pomace. At the moment, the disposal of these waste materials promotes the proliferation of harmful pests, unpleasant odors, and discomfort for producers due to the large generated waste volume. Although open-air disposal is often justified as a method for fertilizing the land, the valuable phytochemicals in grape pomace are overlooked for their potential in value-added applications. This approach ultimately squanders the true potential of grape pomace [13].

The diverse compounds found in grape pomace have enabled its use in a wide range of applications, including skincare products, like creams and sunscreens [12], anticancer and antimicrobial treatments [14], food preservation, meat and fat products [15], and livestock fattening for human consumption [16]. Generally, grape pomace is valued for its ability to prevent oxidative stress. However, as a plant-based material, it is subject to variations, such as climate conditions, terrain differences, variations in pomace production processes, and residue disposal methods. Therefore, it is important to develop strategies and techniques to ensure the homogeneity of grape pomace for future applications.

The aim of this study was to propose a valorization pathway for vine prunings and grape pomace residues generated in Valle de Guadalupe, enabling their use in both energy and non-energy applications. The development of this pathway started with physicochemical characterization, followed by specific applications for each type of residue. For vine prunings, the application considered was direct combustion for energy production. For grape pomace, the proposed application was as an antioxidant agent in vegetable oils. Various tests were performed, including proximate, chemical, elemental, protein, total fat, calorific value, and thermogravimetric (TGA) analyses for both residues. Specifically for grape pomace, the antioxidant capacity was measured using the total phenol content (TPC), ferric reducing antioxidant power assay (FRAP), 2,2-diphenyl-1-picrylhydrazyl assay (DPPH), 2,2′-azino-bis(3ethylbenzothiazoline-6-sulfonic acid assay (ABTS), oxygen radical absorbance capacity (ORAC), total flavonoid content (TFC), and total anthocyanins (TAs). The most representative compounds of the grape pomace extract were identified with high-performance liquid chromatography (HPLC). This extract was evaluated as an antioxidant agent in vegetable oil by measuring the stability oxidative index.

## 2. Results and Discussions

### 2.1. Characterization Results of Grapevine Prunings and Grape Pomace

The complete results for proximate analysis, chemical composition analysis, ultimate analysis, fat content, protein content, and higher heating value are presented in Table 1. The low density of this residue aids in natural drying and moisture loss and presents reactivity and easy ignition. Thus, vine prunings are considered an attractive residue for energy applications, such as direct combustion, pyrolysis, and gasification [17]. In contrast, grape pomace, with its high moisture content and low volatile material content, is not suitable for direct energy applications. Combustion systems require thermal pretreatment to remove water from these samples, reducing the overall energy efficiency. However, grape pomace is suitable for non-energy applications, such as producing reducing sugars, obtaining extracts rich in anthocyanins or antioxidant capacity, and biological degradation processes [18].

Both the vine prunings and grape pomace exhibited similar values in the number of solvent-extractable substances. However, in hot water extractions, the vine prunings showed a higher value compared to the grape pomace. This aligns with the low content of volatile material, as several volatile compounds, such as terpenes, have an affinity for aqueous media, indicating their limited presence [18].

In terms of the lignocellulosic content, the grape pomace had double the value compared to the vine prunings. Conversely, when comparing the holocellulose content, the vine prunings showed higher values, suggesting a more wood-like characteristic in the residue. The calorific value of the grape pomace was 21.85% higher than that of the vine prunings, likely due to the presence of compounds such as sugars and phenolic acids. The calorific value of these residues exceeded the reported range [17,18,22].

The grape pomace contained higher amounts of carbon (C), and hydrogen (H) compared to the vine prunings, which correlates with its higher calorific value, as shown in Table 1. The oxygen (O) content in vine prunings makes them unsuitable for liquid fuel production and increases the generation of combustion products, such as H_2_O, thereby reducing energy efficiency. Nitrogen (N) contributes to NO_x_ emissions, but it is also a crucial element of the protein content. The grape pomace had 4.5 times more nitrogen and 5.5 times more protein than the vine prunings. Regarding sulfur (S), up to 90% was bound or included in the ash content, where the grape pomace exhibited a higher ash percentage compared to the vine prunings. These results indicate that vine pruning applications should be energy-related, such as serving as a raw material for an energy conversion process, like direct combustion [29], while grape pomace is more appropriate for non-energy applications.

### 2.2. Thermogravimetric Analyses Results

**Figure 2:**
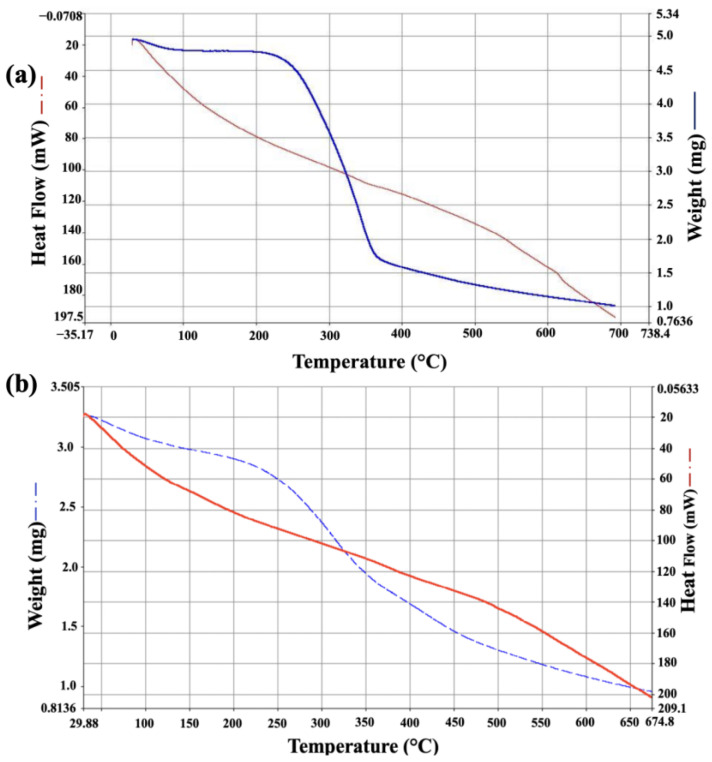
Thermogravimetric results: (**a**) vine prunings; (**b**) grape pomace.

Figure 2 illustrates the TGA of the vine prunings and grape pomace. The blue curve indicates the sample mass, while the red curve represents the energy applied to the sample.

As shown in Figure 2a, the first increment analyzed, ranging from 0 to 100 °C, was related to the evaporation of H_2_O. Once the moisture was removed from the sample, the mass loss remained relatively constant from 100 to 220 °C. After 220 °C, a pronounced decline began, extending up to 350 °C, attributed to the hemicellulose decomposition in the range of 150–310 °C [18]. Above 350 °C and until the end of the test, a constant decline was observed again, indicating cellulose and lignin decomposition between 310 and 400 °C. In Figure 2b, the thermogravimetric analysis of grape pomace began with moisture removal up to 100 °C. The next section, from 100–250 °C, shows the removal of extractable compounds, followed by the hemicellulose decomposition zone between 250 and 350 °C. Beyond this point, two trends were observed: from 350 to 500 °C, cellulose decomposition occurred, and above 500 °C, lignin decomposition predominantly took place in the biomass [18].

### 2.3. Antioxidant Capacity Results

The antioxidant capacity results for samples Ex1–Ex4 are presented in Table 2. Overall, the most effective analyses were achieved using the Ultra-Turrax method, with the citric acid-free mixture yielding the best results for the FRAP, total phenols, and flavonoids assays. The sample containing citric acid showed superior performance in the DPPH, ABTS, ORAC, and anthocyanin tests, demonstrating the synergy of citric acid in the release of anthocyanins.

Compared to the other extracts, Ex5 showed superior results in all assays except for DPPH. It is important to consider the impact of temperature on DPPH assay values, as higher temperatures led to decreased values across all extracts. Ex1 and Ex2 were obtained under 25 °C and demonstrated the highest DPPH values, even higher than those in the literature [20,30,31]. The remaining extracts, Ex3 to Ex5, were obtained at 50 °C, and all of them had a DPPH value below those reported [20,30,31].

In the antioxidant capacity values measured by FRAP and TPC, it can be observed that the type of extraction method played a more significant role than temperature. Using an ultrasonic extraction process did not fully release the phenolic compounds from the grape pomace, but this could be achieved through short-duration grinding. The effect of citric acid played a synergistic role in the determination of radical scavenging by ABTS. When citric acid was combined with the solvent during mechanical extraction, the ABTS capacity increased. However, when the ultrasonic process was used, the capacity decreased. This behavior occurred only in the ABTS assay. In the ORAC determination, introducing citric acid to the mixture increased the μmol of Trolox equivalents, regardless of whether the process was mechanical or ultrasonic.

The TFC was enhanced when using mechanical extractions compared to ultrasound. Additionally, temperature had a significant effect, as the extract with the highest flavonoid content was obtained at 50 °C. For this analysis, no significant effect of time was observed. All the results are within the ranges of those in the literature, except for the anthocyanin content, for which the values in the extracts of grape pomace were below the reported range [20,30,31]. Anthocyanins are susceptible to heat and light degradation; hence, it is crucial to develop an extraction technique that will preserve the extract’s antioxidant potential while preventing the degradation of the anthocyanin content.

The addition of citric acid to extracts Ex2 and Ex4 did not result in an increase in all antioxidant capacity values. At low temperatures, Ex1 showed higher FRAP results than its counterpart, Ex2, which contained citric acid. At 50 °C, extract Ex3 showed higher results than its counterpart, Ex4, which contained citric acid, in the DPPH and ABTS assays. In general, Ex5 showed more balanced results. Increasing the ratio of grape pomace to solvent from 1:10 in Ex1–Ex4 to 2:10 in Ex5 produced higher antioxidant results in all measurements, with the exception of DPPH for Ex3 [37].

### 2.4. HPLC Profiles of Sunflower Oil and Grape Pomace Extract

The fatty acid profile of sunflower oil is shown in Table 3. According to the data, the oil had an omega-6 content of 76.03%, an omega-9 content of 17.28%, and a saturated fatty acid content of 6.69%.

The phenolic acids, anthocyanins, and reducing sugars profile were obtained for Ex5, as it overall yielded the best results in terms of antioxidant capacity and phytochemical content, as can be seen in Table 2. Regarding the acidic compounds, p-coumaric acid was detected at 15.374 mg/L, chlorogenic acid at 7.408 mg/L, caffeic acid at 8.372 mg/L, and ferulic acid at 1.554 mg/L. The values obtained for p-coumaric acid were within the range reported in the literature of 8.00–80.95 mg/kg [21,31]. The amount of caffeic acid was very close to the lower limit reported in the literature, ranging from 11.69 to 116.51 mg/kg [21,31,38]. For both ferulic acid and chlorogenic acid, the values obtained from the extract were below those reported in the literature, with 2.5 mg/kg and 428.3 mg/kg, respectively [31].

In the anthocyanin profile of the Ex5 extract, four compounds were identified: cyanidin at 26.52 mg/L, pelargonidin at 5.25 mg/L, malvidin at 9.86 mg/L, and delphinidin at 9.47 mg/L. The reported ranges in the literature vary between 18.5 and 3597.47 mg/kg for delphinidin, 7.03 and 709.51 mg/kg for cyanidin, and 176 and 10,846.30 mg/kg for malvidin [39,40,41,42,43]. According to the literature, the anthocyanin with the highest concentration in grape pomace extracts is malvidin, with concentrations reaching up to 10,846.30 mg/kg [40]. The authors reported that using processes with 3 h durations and temperatures of 30 °C helped maintain high anthocyanin concentrations in the extracts [40]. The presence of pelargonidin is uncommon in grape pomace extracts, and the value of this anthocyanin in Ex5 was very similar to that reported in the literature [40]. Anthocyanins are predominantly found in the grape pulp, suggesting that residual grape pomace from the wine industry is largely composed of seeds. These findings suggest that the wineries located in Valle de Guadalupe have developed techniques to increase the yields of wine production.

Only two flavonoids were identified in the extracts: catechin at 125.05 mg/L and epicatechin at 214.29 mg/L. The values reported in the literature for catechin in grape pomace extracts range from 46.16 to 390.20 mg/kg [21,31,38]; therefore, the obtained value falls in the middle of the range. For epicatechin, the reported range is from 27.93 to 343.70 mg/kg [21,33], with the value found in this study also within the reported range. This is attributed to the components of grape pomace, as epicatechin is abundant in grape seeds but not in the fruit. Once again, the results suggest that the grape pomace consists largely of seeds, as the grape pulp is used directly in wine production. As the concentration of grape pulp in the pomace was low, so was the sugar content. The reported values for glucose in extracts range from 5.50 to 75.5 g/kg [44,45], compared to 2.810 g/L in the Ex5 extract, while the sucrose content was reported as 16.47 g/kg [45], compared to the content of 4.390 g/L found in this study. These findings may also be explained by the greater content of seeds in the pomace and the improved yields in the local wineries.

### 2.5. Oxidative Stability Index Results

The duration oxidative stability for the sunflower oil was 8 h before degradation began, with conductivity reaching 40 µS∙cm^2^ after 14 h. When grape pomace extract was added to the sunflower oil samples, a decrease in conductivity was observed.

The sunflower oil samples with the grape pomace extract at 720 mg/L, 1473 mg/L, and 2357 mg/L concentrations showed induction times of 10 h, 12 h, and 10.5 h, respectively. The sunflower oil sample with the grape pomace extract at 1473 mg/L revealed the best result, with a 50% OSI increase.

**Figure 3:**
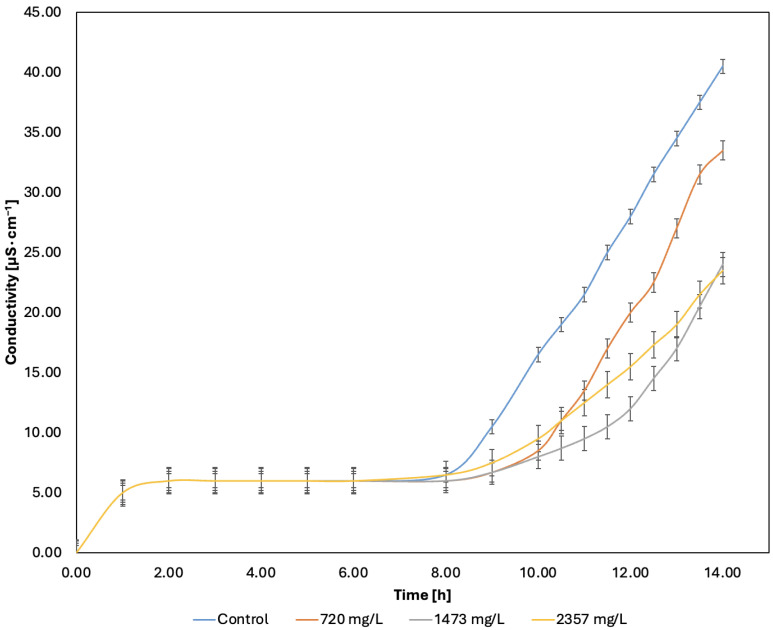
Sunflower oil oxidative stability evaluation.

At 14 h, the lowest conductivity value recorded was 23 µS∙cm^−1^, reflecting a 42.5% reduction from the original sunflower oil value. The four curves are shown in Figure 3.

Daneshniya et al. [46] assessed the oxidative stability of spent grape pomace extracts in sunflower oil. The extraction method used was ultrasound with 50% EtOH, for 30 min, at 10% *w*/*v*. The concentration of the grape pomace extract was 30% *v*/*v*, and it was not concentrated before being added to sunflower oil. The induction time for the control sunflower oil sample was 3.92 h, while for the sample containing the grape pomace extract, it ranged from 4.39 to 4.58 h, an OSI increase of about 16.83%. Comparing the results of Ex5 to those obtained by Daneshniya et al. [46], the present study achieved higher induction times with less extract in sunflower oil.

Gámez et al. [47] evaluated the oxidative stability of grape pomace extract in soybean oil. The extraction method used was maceration with 95% EtOH, for 2 h, at 20% *w*/*v*. The grape pomace extract was concentrated in a rotary evaporator prior to its application and testing in soybean oil. The control soybean oil sample had an induction time of 6.63 h. The grape pomace extracts were tested at 0.02, 0.1, 0.3, and 0.5% *w*/*w* concentrations in oil. The induction times were 10 h, 11 h, 20 h, and 44 h, respectively, highlighting an increase in the OSI from 50.83% to 563.65%. The best result of the present study was found at a concentration of 1473 mg/L, equivalent of 0.15% *w*/*w*, which is similar to the results obtained by Gámez et al. [47] for a concentration of 0.1%. It is important to highlight that the extract in the present study was also concentrated prior to its application in sunflower oil.

The OSI results indicate that grape pomace extract can inhibit the oxidation process of sunflower oil if the extract is concentrated before applying it to the oil, positioning it as a potential natural antioxidant [48].

## 3. Materials and Methods

### 3.1. Sample Collection and Preparation

Vine pruning samples were collected during winter pruning in mid-May, while grape pomace samples were collected in September. Both residues came from the Faculty of Enology and Gastronomy at the Autonomous University of Baja California, Sauzal Campus, Ensenada, Baja California (31.86756292390066, −116.66874469102723) and its local winery partners. The grape pomace came from the grape variety “Cabernet Sauvignon” (*V. vinifera*). The vine prunings were collected with gardener scissors, and the biomass was stored in plastic bags for future analysis. The grape pomace was obtained after wine production; samples were collected in plastic bags and stored in a portable cooler. Both the vine prunings and grape pomace were processed with a manual mill to reduce their size for easier handling, followed by sieving through a No. 35 sieve to ensure uniform particle size. After their pretreatment, the vine prunings and grape pomace samples were stored in a freezer at −6 °C until future analysis.

### 3.2. Proximate Analysis

Following the preparation of the samples, proximate analysis was conducted. A 1 g sample was placed in a pre-weighed dry crucible. The procedure adhered to ASTM standards. The crucibles with the samples were subjected to controlled heating in a muffle furnace. The moisture content was determined using ASTM E871-82 [49], by drying at 105 °C for 6 h until a constant weight was achieved. The volatile matter content was measured according to ASTM E872-82 [50], by drying at 950 °C for 7 min. The ash content was determined using ASTM E830-87 [51], by drying at 580 °C for 4 h. The fixed carbon percentage (%FC) was calculated by subtracting the other components. All determinations were performed in triplicate, and the percentages were calculated based on the weight difference of the crucible before and after each test.

### 3.3. Chemical Composition and Higher Heating Value Analysis

The analysis to determine the extractable content in organic solvent was based on the TAPPI standard [52]. According to this standard, acetone was used for the extraction. A cellulose thimble with a 5 g sample was placed in Soxhlet equipment and boiled for 8 h, with at least 4 siphons per hour. Then, the sample was dried, and the difference in mass was measured using an analytical scale [53].

The second part of the analysis involved the quantification of the extractable content in water. For this test, the TAPPI standard T 207 cm-99 was used [54]. A 4 g dried and acetone-extractable sample was placed in an Erlenmeyer flask with 200 mL of hot water. The flask was placed in a boiling water bath and maintained at a constant volume with a condenser for 3 h. After the test, the content inside the flask was filtered and washed with 200 mL of hot water. Then, the sample was dried, and the difference in mass was measured using an analytical scale [53].

The lignin content was assessed using the standard ASTM D 1106–96 [55]. A 1 g dried sample, obtained after the solvent and hot water extraction, was mixed with 15 mL of 72% H_2_SO_4_ at 13 °C. The mixture was stirred at 2 different steps, first at 400 rpm for 1 min, and then at 200 rpm for 2 h, both speeds at 19 °C. The resulting mixture was transferred to a 1 L Erlenmeyer flask and combined with 560 mL of deionized water. This flask was brought to a constant boil with reflux for 4 h. After the procedure, the sample was cooled to 20 °C, then filtered and washed until a neutral pH was achieved. Finally, the sample was dried, and the difference in mass was measured using an analytical scale [53].

The holocellulose content (%Hol) was determined using the standard ASTM D1104-56 [56]. A 2 g moisture-free and total extractable-free sample was placed in an Erlenmeyer flask with 150 mL of distilled water, 0.2 mL of acetic acid, and 1 g of sodium chlorite, all at 15 °C. A cap was used to close the flask, which was placed inside a water bath at 75 °C for 5 h with constant agitation. During each hour of the bath, 0.22 mL of acetic acid at 15 °C and 1 g of sodium chlorite were added. After 5 h, the flask was cooled down to 10 °C using a cold-water bath. The content was filtered out and washed with cold water until the sample lost its yellow coloration. Then, the sample was dried, and the difference in mass was measured using an analytical scale [53].

The cellulose content determination (%Cel) was performed using a standard ASTM D 1103–60 [57]. A 2 g sample of dried holocellulose was combined with 10 mL of NaOH at 17.5% (*w*/*v*) inside an Erlenmeyer flask. The mixture was left to cool for 5 min at 20 °C. Every 5 min, another 5 mL of NaOH at 17.5% (*w*/*v*) was added to the flask, until a total of 25 mL of NaOH was in the flask. After this, the sample was left to rest for 30 min. The analysis continued with the addition of 33 mL of deionized water at 20 °C, and the sample was left to rest for another 1 h. The total duration of the test was 105 min. Then, the flask contents were filtered and washed first with 100 mL of NaOH at 8.3% (*w*/*v*), then with deionized water, followed by acetic acid at 10% (*v*/*v*), and finally with deionized water until a neutral pH was reached. The sample was dried, and the difference in mass was measured using an analytical scale. Finally, the percentage of hemicellulose (%Hem) was measured by subtracting the percentage of cellulose (%Cel) from the percentage of holocellulose (%Hol) [53].

The higher heating values (HHV) of the vine prunings and grape pomace were determined in duplicate by weighing 0.5 g of each sample. The experiment was conducted using an IKA C2000 bomb calorimeter, following the methodology outlined in ASTM 711 [58].

### 3.4. Elemental, Thermogravimetric, Protein, and Fat Analyses

Elemental analysis of the vine prunings was performed at the Faculty of Chemistry, National University of Colombia, under the following conditions: a Thermo Flash 2000 ECHNS-O analyzer by Thermo Scientific (Waltham, MA, USA) was used, with the reactor set to 900 °C. Vanadium oxide was used as a catalyst for the CHNS reactor at 1060 °C for oxygen analysis. For the grape pomace, the analysis was conducted at the Faculty of Chemistry, UNAM, in the Support Services Unit for Research and Industry (U.S.A.I.I.), using a Perkin Elmer (Waltham, MA, USA) elemental analyzer model PE2400, helium as the carrier gas, and a thermal conductivity detector. The combustion reactor temperature was 975 °C, and the reduction reactor temperature was 501 °C.

In the thermogravimetric analysis, ground and sieved samples were analyzed using a Perkin Elmer STA 6000 differential thermal analyzer. This process was conducted to observe the decomposition reactions and devolatilization behavior of the materials [18].

To determine the fat content, 2 g of grape pomace was weighed and placed in pre-dried and weighed cellulose thimbles. These were then transferred to a Soxhlet extractor, with enough hexane added for 2 to 3 cycles. The extraction process was conducted for 6 h. After extraction, the thimbles were dried at 100 °C until a constant weight was achieved. The fat content was determined by the weight difference [19]. For protein determination, a 0.2 g sample was accurately weighed and placed in a flask with the addition of 3 mL of concentrated H_2_SO_4_. The mixture was immediately heated in a Digesdahl digestion apparatus at 430 °C for 3 min, ensuring the sample did not dry out. Subsequently, 10 mL of refrigerated 50% H_2_O_2_ was added, and the reaction was allowed to proceed for an additional 2 min or until the effervescence ceased. The digestion flask was then removed and allowed to cool for 15 min before being filled with deionized water. A 1 mL aliquot of the solution was transferred to a 50 mL volumetric flask. The samples were then prepared for subsequent analysis of the total nitrogen content as NO_3_ at 460 nm using a Hach (Singapore) DR 5000 spectrophotometer.

### 3.5. Extraction Elaboration

Grape pomace extracts were prepared from dried plant material. The drying process was conducted in a muffle furnace at 65 °C for 3 days. Four extracts were prepared from this dried material, maintaining a consistent 1:10 ratio of plant material to solvent. For the first extract (Ex1), 4 g of dried grape pomace was weighed and mixed with 40 mL of a solvent composed of 55% ethanol and 45% water. The mixture was then homogenized using an Ultra-Turrax (Singapore) IKA T18 for 2 min. The second extract (Ex2) was prepared under the same conditions, but with a different solvent composition: 50% ethanol, 48% water, and 2% citric acid; the latter has been shown to enhance the anthocyanin content in plant extracts [2,10,16]. For the third extract (Ex3), 4 g of dried grape pomace was weighed and mixed with 40 mL of a mixture of 55% ethanol and 45% water. The extract was then subjected to ultrasonic treatment using an Elmasonic Easy device for 30 min at 50 °C. The fourth extract (Ex4) was prepared in a similar manner to Ex3, with the only variation being the type of solvent used: 50% ethanol, 48% water, and 2% citric acid; once again, the citric acid addition was to improve the anthocyanin content in the grape pomace extract [2,10,16]. Based on the results of extracts Ex1–Ex4, a fifth extract (Ex5) was prepared to enhance the antioxidant capacity, avoiding the addition of citric acid. This was achieved by using a solvent of 55/45% ethanol/water, a ratio of grape pomace to solvent of 2:10, and Ultra-Turrax grinding for 2 min at 50 °C.

### 3.6. Antioxidant Capacity and Phytochemical Content

#### 3.6.1. Total Phenolic Content (TPC)

This procedure measures the ability of phenols to reduce molybdenum VI to molybdenum V. For this, a 15 µL sample was mixed with 37 µL of Folin Ciocalteu reagent, 128 µL of H_2_O, and 120 µL of sodium carbonate at 7.1% *w*/*v* [31,59]. The reaction time for the mixture was 60 min, and then the absorbance was measured in a Multiskan Spectrum (Thermo-Scientific, Waltham, MA, USA) at 760 nm. The determination was performed by comparing the results to a standard curve of gallic acid, and the concentration was expressed in mg of gallic acid per 100 g of sample. The experiment was conducted in triplicate, and the average result is presented in Table 2 with the standard deviation.

#### 3.6.2. Ferric Reducing Antioxidant Power (FRAP) Assay

This method evaluates the ability of a sample to reduce the complex ferric iron (Fe^3+^) 2,4,6-Tris(2-pyridyl)-*s*-triazine (TPTZ) to the ferrous form (Fe^2+^), which has a visible band at 593 nm [20,60]. A working solution for FRAP was prepared by mixing 1 mL of TPTZ solution at 10 mmol/L, 1 mL of FeCl_3_ at 20 mmol/L, and 10 mL of an acetate buffer at pH 3.4. A sample aliquot of 15 µL was combined with 285 µL of the working solution. The mixture was incubated for 30 min, and the absorbance was measured in a Multiskan Spectrum (Thermo-Scientific) at 593 nm. For the determination, a standard curve of ascorbic acid was used as the reference, and the results are expressed as mg of ascorbic acid equivalent per 100 g of extract. The experiment was conducted in triplicate, and the average result is presented in Table 2 with the standard deviation.

#### 3.6.3. ABTS Scavenging

The ABTS^+^ cation radical was generated through the oxidation of ABTS by ammonium persulfate in a phosphate buffer at pH 7.4 [60]. An aliquot of 15 μL was taken from the sample and mixed with 285 µL of the ABTS^+^ solution. The mixture was left to react for 30 min at room temperature, and the absorbance was measured in a Multiskan Spectrum (Thermo-Scientific) at 734 nm. For the determination, a standard curve of Trolox as a reference was used, and the results are expressed as µmol of Trolox equivalent per 100 g of extract [60]. The experiment was conducted in triplicate, and the average result is presented in Table 2 with the standard deviation.

#### 3.6.4. Total Flavonoid Content (TFC)

The method used was based on the procedure proposed by Corrales [61], with some modifications. In an Eppendorf tube, a sample aliquot of 30 µL was mixed with 114 µL of water, 15 µL of 5% NaNO_2_ (*w*/*v*), 15 µL of 10% *w*/*v* AlCl_3_, 60 µL of 1 M NaOH, and 66 µL of H_2_O. The absorbance was measured in a Multiskan Spectrum (Thermo-Scientific) at 510 nm. For the determination, a standard curve of (+)-catechin as a reference was used, and the results are expressed in mg of catechin equivalent per 100 g of sample. The experiment was conducted in triplicate, and the average result is presented in Table 2 with the standard deviation.

#### 3.6.5. ORAC Assay

This method is based on the protection given by a sample to fluorescein when being oxidized by free radicals [33]. A sample of 30 μL was mixed with 2920 μL of a fluorescein solution at 70 nM using a phosphate buffer as a solvent. The concentration of the buffer was 75 nM and it had a pH of 7.4. The cell was placed in the sample holder of a LS55 Fluorescence Spectrometer (Perkin Elmer, Waltham, MA, USA). Then, 50 µL of an AAPH solution at 0.6 mol/L was added. A control was prepared following the same procedure, without adding the sample. The excitation and emission wavelengths were 493 nm and 515 nm, respectively. The software Origin Pro 8 (OriginLab Corporation, Northampton, MA, USA) was used to measure the area under the curve (AUC) with the kinetic data provided by the fluorescence spectrometer. The ORAC value was obtained using Equation (1), with the results are expressed in μmol of Trolox equivalent per 100 g of sample.

(1) $$\mathrm{ORAC}=\frac{\mathrm{AUC}\;-\;\mathrm{AU}C^{{}^{\circ}}}{{\mathrm{AUC}}_{\mathrm{Trolox}}\;-\;\mathrm{AU}C^{{}^{\circ}}}\rho$$

where AUC is the area under the curve of the sample, AUC° is the control area under the curve, AUC_Trolox_ is the area under the curve for Trolox, and ρ is the ratio between Trolox and the sample concentrations. The experiment was conducted in triplicate, and the average result is presented in Table 2 with the standard deviation.

#### 3.6.6. DPPH Free Radical Scavenging

The antioxidant activity of the samples was evaluated based on the consumption of DPPH, according to the method proposed by Alzate-Arbeláez, with some modifications [59]. A sample volume of 15 μL was mixed with 285 μL of DPPH solution at 0.2 mM using methanol as the solvent. The mixture was left to react for 30 min in a dark place. Then, the absorbance was measured in a Multiskan Spectrum (Thermo-Scientific) at 517 nm. For the antioxidant activity, the results were compared with Trolox and expressed in µmol of Trolox equivalent per 100 g of sample. The experiment was conducted in triplicate, and the average result is presented in Table 2 with the standard deviation.

#### 3.6.7. Total Anthocyanin (TA) Content

The total anthocyanin content was determined by a differential pH measure [62]. For this procedure, sample aliquots of 100 µL were needed. The first one was mixed with 900 µL of a buffer made of potassium chloride at 25 nM, with the pH adjusted to 1 using a 37% HCl solution. The second was mixed with 900 µL of buffer made of sodium acetate at 0.4 M, with the pH adjusted using a 37% HCl solution until it reached a pH of 4.5. The 2 samples were measured at 530 nm and 700 nm, respectively, with a Multiskan Spectrum spectrophotometer (Thermo Scientific). The total anthocyanin content was expressed as cyanidin 3-glucoside equivalents, in mg per 100 g of sample, calculated using Equation (2).

(2) $$\frac{mgC3G}{100g}=\frac{\left[{\left(A_{530}-A_{700}\right)}_{pH=1}-{\left(A_{530}-A_{700}\right)}_{pH=4.5}\right]\left(MW\right)\left(1000\right)}{\left(\varepsilon )(L)(C\right)}$$

where A represents the absorbance measured at a given wavelength and pH, MW is the molecular weight of cyanidin-3-glucoside (449.2 g/mol), ε is the molar extinction coefficient of cyanidin-3 glucoside at 26,900 L/(mol cm), L is the pathlength (1 cm), and C is the concentration of the extract in g/L. The experiment was conducted in triplicate, and the average result is presented in Table 2 with the standard deviation.

### 3.7. HPLC Profile of Grape Pomace Extract and Sunflower Oil

#### 3.7.1. Phenolic Acids

The determination of phenolic compounds was performed using calibration curves of chlorogenic acid, p-coumaric acid, caffeic acid, and ferulic acid, using liquid chromatography with a diode arrangement detector (HPLC-DAD, Shimadzu^®^, Tokyo, Japan) [63]. Before injection, the samples were filtered with a pore size of 0.45 µm, the wavelength used was 280 nm, and the stationary phase was an aqueous LiChrospher RP-18 column (particle size of 5 μm, 250 mm in length, and 4.6 mm in diameter, Merck, Boston, MA, USA). The mobile phase was a mixture of formic acid at 0.1% (solvent A) and acetonitrile (solvent B), using the following stages: 5% solvent B from min 0 to 16; 30% solvent B at min 17; 70% solvent B from min 18 to 19, 80% solvent B at min 20; and 5% solvent B from 21 to 30 min. The mobile phase flow rate was 1.0 mL/min at 45 °C. The results are expressed in mg of acid per 100 g of sample.

#### 3.7.2. Catechin and Epicatechin Determination

The (+)-catechin and (−)-epicatechin contents were measured by liquid chromatography (HPLC-DAD, Shimadzu®), using calibration curves for both [64]. The samples were filtered through 0.40 μm filters into vials, and then injected into a chromatographer equipped with a SIL-20A/HT auto-injector, a CBM-20A communication module, and an SPD-M20A photodiode array detector (PDA). Calibrating to 280 nm, (+) catechin/(−) epicatechin quantification was performed in a C18 column with the following dimensions: particle size of 5 μm, 250 mm in length, and 4.6 mm in diameter. The mobile phase for elution was a mixture of 0.1% formic acid (solution A) and acetonitrile (solution B) delivered at a flowrate of 1.0 mL/min. The gradient used had the following stages: 5% solvent B for min 0–30; 35% solvent B for min 31–34; 35% solvent B for min 36, and finally, 5% solvent B for min 36–40. The results are expressed in mg of acid per 100 g of sample.

### 3.8. Oxidative Stability Index of Sunflower Oil

The extract demonstrating the highest antioxidant capacity was selected for encapsulation and subsequent application in a sunflower oil without any kind of antioxidants in it. Ethanol was removed from the extract via rotary evaporation, reducing 90 mL of the original extract, comprising 45% water and 55% ethanol, to a final volume of 39 mL. The concentrated extract was then mixed at a 1:1 ratio with polyethylene glycol, stirred, and stored in amber containers inside a freezer at −6 °C.

The fatty acid profile for the sunflower oil used was determined using an Agilent (Santa Clara, CA, USA) 6890N Gas Chromatograph equipped with a 5973N Mass Selective Detector and a DB1-MS column. The analysis was conducted under the following conditions: Helium was used as the carrier gas in split mode. A 0.2 μL injection volume was employed, with both the injector and detector maintained at 250 °C. The oven temperature program began at 100 °C, held for 1 min, then increased at 5.00 °C/min to 150 °C, held for 2 min, followed by an increase at 5.00 °C/min to 250 °C, and held for 3 min. The final ramp increased the temperature at 10.00 °C/min to 300 °C, where it was held for 5 min. The total run time was 59.81 min [65].

The grape pomace extract was retrieved from the freezer and stored in a dark place until it reached an ambient temperature. Then, 4 tubes were prepared for the oxidative stability index (OSI) test. The first tube contained pure sunflower oil as a control, tubes 2–4 contained sunflower oil with different concentrations of the grape pomace extract, the values of which were 720 mg/L, 1473 mg/L, and 2357 mg/L. Each tube contained an aliquot of 2.5 g of sunflower oil. The OSI test was performed by the Rancimat model 679 (Metrohm, Herisau, Switzerland) following the OCS Official Method Cd 12b-92 [66]. The aliquots of sunflower oil were heated to 120 °C and oxidized by bubbling air through the sample (20 L·h^−1^). Volatile secondary products from lipid oxidation were carried by the air into a tube with distilled water, where the conductivity was monitored. The OSI was recorded as the time until the maximum rate of conductivity increase was achieved [65].

## 4. Conclusions

Vine pruning and grape pomace waste originating from Valle de Guadalupe present an opportunity to create a biomass waste valorization pathway by obtaining value-added products across two distinct applications. Vine prunings are a viable material for use as firewood and other combustion applications for energy due to their low extractable content, chemical composition, and higher heating value. On the other hand, the grape pomace extracts exhibited antioxidant capacity and the presence of phytochemical compounds. The selected extract was obtained through Ultra-Turrax grinding at 50 °C with ethanol and water for 2 min and a ratio of 2:10 grape pomace/solvent. The anthocyanin profile of Ex5 showed concentrations of cyanidin at 26.52 mg/L, pelargonidin at 5.25 mg/L, malvidin at 9.86 mg/L, and delphinidin at 9.47 mg/L. The catechin content at 125.05 mg/L and the epicatechin content at 214.29 mg/L, along with the low content of anthocyanins, suggest that the pomace contained a higher proportion of seeds than pulp. This information highlights that the wine industry maximizes the use of pulp in wine production. Although there was still 2.810 g/L of glucose and 4.390 g/L of sucrose in the grape pomace samples, it is recommended to assess their potential for reuse in the creation of low-cost wines. Grape pomace functions as an antioxidant agent in sunflower oil. According to the oxidative stability results, the addition of grape pomace extract increased the induction time up to 50%. These results provide a comprehensive set of analyses and techniques that are not found in the literature and offer evidence to create a valorization pathway for vine prunings and grape pomace generated in Valle de Guadalupe.

## Figures and Tables

**Table 1:** Proximate, chemical, ultimate, protein, fat, and higher heating value analysis results (d.w. = dry weight).

Parameter	Grapevine Prunings			Grape Pomace		
	Value	Other Authors	Reference	Value	Other Authors	Reference
Humidity (%)	9.14 ± 0.14	9.24	[17]	63.2 ± 0.22	59.81–72.73	[19,20,21]
Volatile material (%)	73.05 ± 0.18	66.29	[17]	79.64 ± 0.12	62.07–70.6	[18,22,23]
Ashes (%)	3.70 ± 0.69	12.36	[17]	7.36 ± 0.26	0.65–8.3	[18,22,23]
Fixed carbon (%)	23.25 ± 0.62	12.12	[17]	13.34 ± 0.27	25.6–30.95	[19,20,21]
Water extractables (%)	11.46 ± 0.54	14.2	[24]	11.29 ± 0.49	16.65	[18]
Solvent extractables (%)	19.72 ± 0.34	-	-	13.22 ± 0.62	1.63	[18]
Lignocellulose (%)	17.66 ± 1.07	41.10–90.00	[25,26]	36.94 ± 1.18	46.70–63.00	[25,26,27]
Holocellulose (%)	78.06 ± 0.71	-	-	58.70 ± 0.83	21.45–45.00	[18,28]
Cellulose (%)	48.68 ± 0.056	-	-	16.76 ± 0.44	12.9–19.00	[18,28]
Hemicellulose (%)	29.41 ± 0.86	-	-	41.93 ± 0.72	8.55–26.00	[18,28]
HHV (MJ/kg)	17.02 ± 0.26	14.36	[17]	20.74 ± 0.33	18.76–19.67	[18,22,23]
Protein (%/g d.w.)	5.51 ± 0.19	-	-	30.48 ± 0.38	1.30–10.04	[19,20,27]
Fat content (%)	-	-	-	4.485 ± 0.11	3.67	[19]
C (%)	43.07	39.88–46.20	[17,18,19,20,21,22,23,24]	49.32	49.10–53.71	[18,22,23]
H (%)	5.31	7.16	[17,18,19,20,21,22,23,24]	6.07	5.14–6.28	[18,22,23]
O (%)	40.93	0.16	[17,18,19,20,21,22,23,24]	-	33.33–38.46	[18,22,23]
N (%)	0.78	0.60–1.86	[17,18,19,20,21,22,23,24]	3.54	1.17–2.94	[18,22,23]
S (%)	-	38.83	[17,18,19,20,21,22,23,24]	0.55	0.04–1.15	[18,22,23]

**Table 2:** Antioxidant capacities of grape pomace extracts.

Analysis	Current Work					Other Authors	
	Ex1	Ex2	Ex3	Ex4	Ex5	Range	Reference
DPPH (μmol Trolox/100 g sample)	8699.17 ± 745.56	9180.13 ± 46.46	1762.06 ± 129.17	1278.96 ± 64.15	1704.41 ± 16.78	3355–6000	[20,30,31]
FRAP (mg ascorbic acid/100 g sample)	2003.45 ± 88.79	1551.33 ± 79.06	474.6 ± 23.33	553.92 ± 42.44	2179.19 ± 36.51	402–649	[20,30,32]
TPC (mg gallic acid/100 g sample)	1261.42 ± 12.48	1220.28 ± 26.71	350.98 ± 17.99	470.3 ± 17.13	1668.10 ± 4.76	386.62–8700	[20,30,31,32]
TFC (mg Eq. catechin/100 g sample)	589.07 ± 31.78	545.07 ± 28.45	210.49 ± 8.90	289.63 ± 8.63	1330.39 ± 43.92	742–2632	[30,31,33]
ABTS (μmol Trolox/100 g sample)	31,636.15 ± 2000.97	42,813.45 ± 2433.87	12,026.03 ± 541.57	6093.58 ± 409.10	48,271.31 ± 1544.47	3176.32–2573.80	[20,34,35]
ORAC (μmol Trolox/100 g sample)	28,724.52 ± 2016.07	34,554.21 ± 1987.52	8565.85 ± 608.34	13,329.37 ± 23.14	53,694.93 ± 1524.28	3641–76,854.80	[32,33,36]
Anthocyanins (mg cyanidin 3-glucoside/100 g sample)	7.04 ± 1.79	7.5 ± 0.29	4.67 ± 0.76	6.063 ± 0.19	12.61 ± 1.59	136.83–837	[20,30,31]

**Table 3:** Sunflower oil fatty acid profile.

PK	RT	Area Pct	Library ID	Ref	CAS	Type
1	17.7982	6.5918	Hexadecanoic acid, methyl ester	115,367	000112-39-0	Saturated
2	20.5202	44.5955	9,12-Octadecadienoic acid (*Z*,*Z*)-, methyl ester	116,129	000112-63-0	Omega-6
3	20.6523	28.4879	7-Octadecenoic acid, methyl ester	13,049	057396-98-2	Omega-6
4	21.0399	6.2836	Octadecanoic acid, methyl ester	28,371	000112-61-8	Omega-9
5	23.9116	0.4666	Eicosanoic acid, methyl ester	115,427	001120-28-1	Omega-9
6	26.5455	1.5713	Docosanoic acid, methyl ester	115,474	000929-77-1	Omega-9
7	29.4348	0.776	Eicosanoic acid, methyl ester	140,310	001120-28-1	Omega-9
8	33.1874	1.8384	1-Docosene	129,889	001599-67-3	Omega-6
9	38.9837	2.8077	2-(2-Bromoethyl)cyclohexanone	60,261	1000195-45-9	Omega-9
10	46.7708	5.1199	2-Dodecylcyclobutanone	83,998	035493-46-0	Omega-9
11	53.0603	1.4612	Carbonic acid, methyl tridecyl ester	97,434	1000314-62-4	-

## Data Availability

The authors will share any data regarding the findings upon request.
